# Neuroendocrine neoplasms of the stomach. Update on diagnostic criteria, classification, and prognostic markers

**DOI:** 10.1007/s00428-025-04340-x

**Published:** 2025-11-22

**Authors:** Silvia Uccella, Stefano La Rosa

**Affiliations:** 1 Department of Biomedical Sciences, Humanitas University, Milan, Italy; 2 Pathology Service, Istituti di Ricovero e Cura a Carattere Scientifico (IRCCS) Humanitas Research Hospital, Milan, Italy; 3 Unit of Pathology, Department of Medicine and Technological Innovation, University of Insubria, Via Guicciardini 9, 21100 Varese, Italy; 4 Hereditary Cancer Research Center, Department of Medicine and Technological Innovation, University of Insubria, Varese, Italy; 5 Unit of Pathology, ASST dei Sette Laghi, Varese, Italy

**Keywords:** Stomach, Neuroendocrine tumor, Neuroendocrine carcinoma, MiNEN, Classification, Prognosis

## Abstract

Gastric neuroendocrine neoplasms (gNENs) encompass all the spectrum of NENs including neuroendocrine carcinomas (gNECs), mixed neuroendocrine/non-neuroendocrine neoplasms (gMiNENs) and neuroendocrine tumors (gNETs). Differently from other digestive sites, gNETs are subclassified according to the clinicopathologic setting in which they arise, with important prognostic implications. Since gastric endoscopic biopsies represent a high-volume daily activity in both referral and community hospitals, pathologists should be increasingly aware of this disease. This will allow to correctly identify the different entities and, when present, their precursor lesions, giving useful information to clinicians for the best patient management. This review paper aims to provide morphologic, immunophenotypic, and, when necessary, molecular criteria for the correct diagnosis and subtyping of gNENs. In addition, a simplified prognostic classification schema of enterochromaffin-like (ECL)-cell NETs is proposed, based on gastrin serum levels and the status of gastric acid secretion.

## Introduction

The incidence of gastric neuroendocrine neoplasms (gNENs) has been increasing over the last 30 years [1–4] and their classification has evolved to encompass a wide clinicopathologic spectrum [5]. The correct diagnosis is crucial for appropriate patient management that may include radically different strategies from simple endoscopic monitoring to surgery or chemotherapy [6, 7].

Given these premises and considering the widespread use of gastric endoscopy in both referral and community hospitals, it is essential that pathologists are aware of the diversity of gNENs and can accurately distinguish among the different entities in order to provide relevant information for optimal patient management.

This review aims to provide the morphologic, immunophenotypic, and, where appropriate, molecular criteria necessary for the accurate diagnosis of gNENs. In addition, a simplified prognostic classification of enterochromaffin-like (ECL)-cell NETs is proposed, based on gastrin serum levels and the status of gastric acid secretion.

## Classification of gastric NENs (gNENs)

According to the last WHO classification of digestive tumors [5], gNENs are classified into three different categories including neuroendocrine tumors (gNETs), neuroendocrine carcinomas (gNECs) and mixed neuroendocrine-non-neuroendocrine neoplasms (gMiNENs) (Table 1). Notably, gNETs are peculiar — if not unique — among NETs, as they exhibit specific site-related features that must be considered to provide a clinically meaningful classification. For this reason, the WHO classification of NETs into three proliferative grades (NET G1, G2, and G3) should be interpreted in conjunction with clinicopathologic features specific to the gastric setting. In contrast, the criteria for diagnosing NECs and MiNENs are consistent across anatomical sites and these cancers share the morphologic, immunophenotypic, molecular, clinical, and prognostic features of NECs and MiNENs arising in other locations.

**Table 1:** WHO classification of digestive neuroendocrine neoplasms

Tumor category	Morphology	Mitotic index (mitoses/2 mm^2^)	Ki67 index
NET G1	Well differentiated	< 2	Ki67 < 3%
NET G2	Well differentiated	2–20	3–20%
NET G3	Well differentiated	> 20	> 20%
NEC	Poorly differentiated	> 20	> 20%
MiNEN	Well or poorly differentiated	*	*

*NET*, neuroendocrine tumour; *NEC*, neuroendocrine carcinoma; *MiNEN*, mixed neuroendocrine-non-neuroendocrine neoplasm; *: variable depending on the neuroendocrine component type

### Gastric NETs (gNETs)

In normal gastric mucosa, different neuroendocrine cell types are present, some of them showing specific topography, related to their function. Gastrin-producing cells (G-cells) are typically located in the antrum; histamine-producing enterochromaffin-like cells (ECL-cells) are scattered in the oxyntic mucosa of the corpus-fundus; other cell types, among which somatostatin-producing cells (D-cells) and serotonin-producing enterochromaffin cells (EC-cells) are the most represented, are widely distributed [8]. This variety of cells reflects the multiplicity of gNET types and is also related to their pathogenesis and specific site of occurrence. Indeed, the most common gNET types are, in order of frequency, ECL-cell NETs, exclusively arising in the oxyntic mucosa, G-cell NETs, typically found in the antro-pyloric region, and EC-cell NET, that can be observed in both antral and corpus/fundus mucosa [1].

The estimated annual incidence of gNETs, considered all together, is about 0.5 cases/100,000 person, with a female predominance and the mean patient’s age at diagnosis is 64 years. However, it is worth noting that some epidemiological features including gender distribution or age at diagnosis differ among tumor subtypes [1, 9].

#### ECL-cell NETs

ECL-cell NETs are the most frequent type, accounting for more than two-thirds of all gNENs and represent a multifaceted tumor type, with important pathogenetic and clinical correlates.

**Fig. 1:**
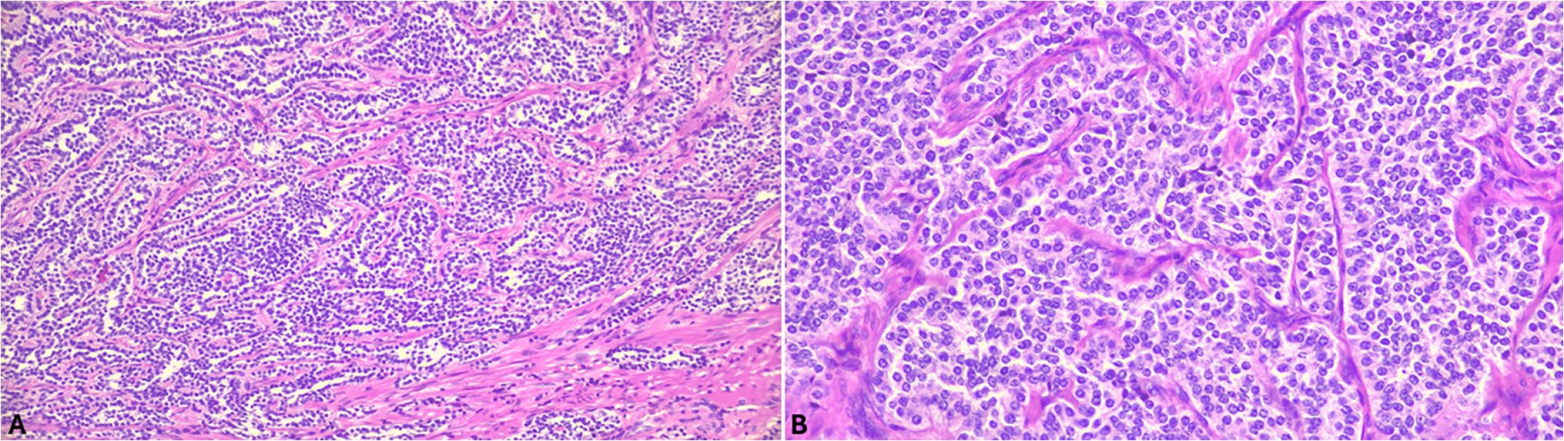
Low power magnification of a gastric ECL-cell NET showing a microlobular and trabecular pattern of growth (**A**). At higher magnification, tumor cells appear uniform cells with monomorphic round nuclei lacking prominent nucleoli and moderately abundant eosinophilic cytoplasm (**B**)

At the histopathologic level, ECL-cell NETs are characterized by organoid architecture, frequently nested and/or trabecular, and are composed of uniform cells showing monomorphic round nuclei with the typical “salt and pepper” chromatin distribution, small to absent nucleoli and moderate amounts of granular eosinophilic cytoplasm (Fig. 1). Most of ECL-cell NETs are grade 1 or 2, thus necrosis is not a feature and mitoses are rare [1]. In addition to these typical morphologic features, unconventional aspects have recently been described in up to 30% of ECL-NETs and should be considered, as they may represent a diagnostic challenge [10]. The most common unconventional pattern (about 60% of unconventional morphologies) is represented by a cribriform network of atrophic-appearing tumor cells embedded in myxoid matrix (*secretory-cribriform variant*). A proportion of cases (31%) shows sheets of bland non-cohesive cells resembling inflammatory elements (*lympho-plasmacytoid variant*). Lastly, a minority of cases (14%) features wreath-like arrangement of columnar cells wrapped around collagenous cores (*pseudopapillary variant*) [10]. In addition, pancreatic acinar cell differentiation, demonstrated by using acinar cell markers (trypsin and BCL10) can be rarely observed [11]. Like all other digestive NETs, gNETs must be graded according to the mitotic index and Ki67 proliferation index. By immunohistochemistry, tumor cells are positive for low molecular weight cytokeratins (suggested are CK8/18 and CAM 5.2), synaptophysin, chromogranin A, insulinoma-associated protein 1 (INSM1), vesicular monoamine transporter 2 (VMAT2), histidine decarboxylase (HDC), and somatostatin receptor type 2 A (SSTR2A). In addition, they may also show scattered cells positive for serotonin, ghrelin, and somatostatin [1].

Despite these shared histopathologic features, ECL-cell NETs are not a monolithic entity. Back in 1993, Rindi et al. distinguished three distinct ECL-cell NET types with different clinical and prognostic meaning, depending on the associated clinicopathologic background [12]. Namely, a type 1, related to autoimmune atrophic gastritis, a type 2, related to Zollinger Ellison syndrome, and a type 3 unrelated to any other pathologic condition (so-called “sporadic”) were recognized [12]. Over time, it has become evident that ECL-cell NETs can be effectively classified considering a panel of histopathologic, biochemical, serologic, genetic and clinical aspects. Specifically, ECL-cell NETs are now classified into 5 types based on the morphology of peritumoral oxyntic mucosa (atrophic, hyperplastic or normal), the presence or absence of antral G-cells hyperplasia, the levels of circulating gastrin, the presence of hypo/achlorhydria, the presence of autoantibodies directed against parietal cells and/or intrinsic factor, the presence of megaloblastic anemia, the setting of MEN1 or other genetic defects, and the assumption of proton pump inhibitor drugs [13] (Table 2).

**Table 2:** Clinico-pathological features of gastric NETs

	M:F ratio	%	MEN1 syndrome	Hypergastrinemia	Acid secretion	Peritumoral mucosa	ECL-cell proliferations	Antral G-cell hyperplasia	Grading	Metastasis	5-year survival
Type 1 ECL-cell tumor	1:2.5	80–90%	No	Yes	Low or absent	Chronic atrophic gastritis	Yes	Yes	-G1 -G2, rare -G3, very rare	1–5%	about 100%
Type 2 ECL-cell tumor	1:1	1%	Yes	Yes	High	Parietal cell hyperplasia	Yes	No	-G1 -G2 -G3, rare	10–30%	60–90%
Type 3 tumor	2.8:1	10–15%	No	No	Normal	Normal	No	No	-G1: 46% -G2: 37% -G3: 17%	40–60%	55–80%
Type 4 ECL-cell tumor	0.5:1	Rare	No	Yes	Low or absent	- oxyntic gland hyperplasia - cystic dilatation - intraluminal eosinophilic material - vacuolated parietal cells	Yes	Yes	-G1 -G2	Rare	Unknown
Type 5 ECL-cell tumor	1.2:1	5–10%	No	Yes	Low or absent	- oxyntic gland dilatation - parietal cell with apocrine-like swelling - cytoplasm snouting	Yes	Yes	-G1 -G2 -G3, rare	10%	about 100%
Gastrin-producing tumor	Unknown	Very rare	No	Possible	Possible high	Normal or gastritis	Not reported	No	-G1 -G2	Rare	Unknown
Somatostatin producing tumor	Unknown	Very rare	No	No	Normal	Normal or gastritis	Not reported	No	-G1 -G2	Rare	Unknown
Serotonin-producing tumor	Unknown	Very rare	No	No	Normal	Normal or gastritis	Not reported	No	-G1 -G2 -G3, rare	Rare	Unknown

In this context, since the different ECL-cell NET types have different prognostic implications [13], it clearly appears that the simple evaluation of tumor morphology is not sufficient to correctly classify ECL-cell tumors. For this reason, in absence of evaluable peritumoral mucosa and of a complete clinicopathologic assessment, the pathology report should only be provisional. A note stating the need for additional specific information should be included. This approach may appear pedantic but is of crucial clinical relevance because the different ECL-cell tumor subtypes show different clinical courses and need different clinical management.

#### Type 1 ECL-cell NET

Type 1 ECL-cell NETs are the most common subtype of gNETs, accounting for about 80–90% of all ECL-cell NETs [1, 4]. They are pathogenically related to atrophic gastritis of the oxyntic mucosa. This condition, related to hypo/achlorhydria, causes an hyperstimulation of antral gastrin-producing G-cells, with consequent hypergastrinemia. The target cells of gastrin in the gastric mucosa are histamine-producing ECL-cells, which begin to proliferate due to the unopposed gastrin stimulation. This condition is primarily related to autoimmune chronic atrophic gastritis (ACAG), although *Helicobacter pylori* infection has also been implicated in some cases [7].

Endoscopically, about 60% of cases present as multiple small (77% < 1 cm, 97% < 1.5 cm) polyps or nodules located in the oxyntic mucosa. Usually, they are confined to mucosa and submucosa with only rare cases, usually larger than 1 cm, showing infiltration of the muscular layer, a morphological feature increasing the risk of lymph node metastasis development [14]. Most cases are grade 1 (G1) or grade 2 (G2) [15], but rare G3 type 1 ECL-cell NETs have been described as well [16]. The Ki67-proliferation index does not seem to have a significant prognostic impact in type 1 ECL-cell tumors, whereas the deep infiltration of the gastric wall seems to be the most important prognostic factor.

**Fig. 2:**
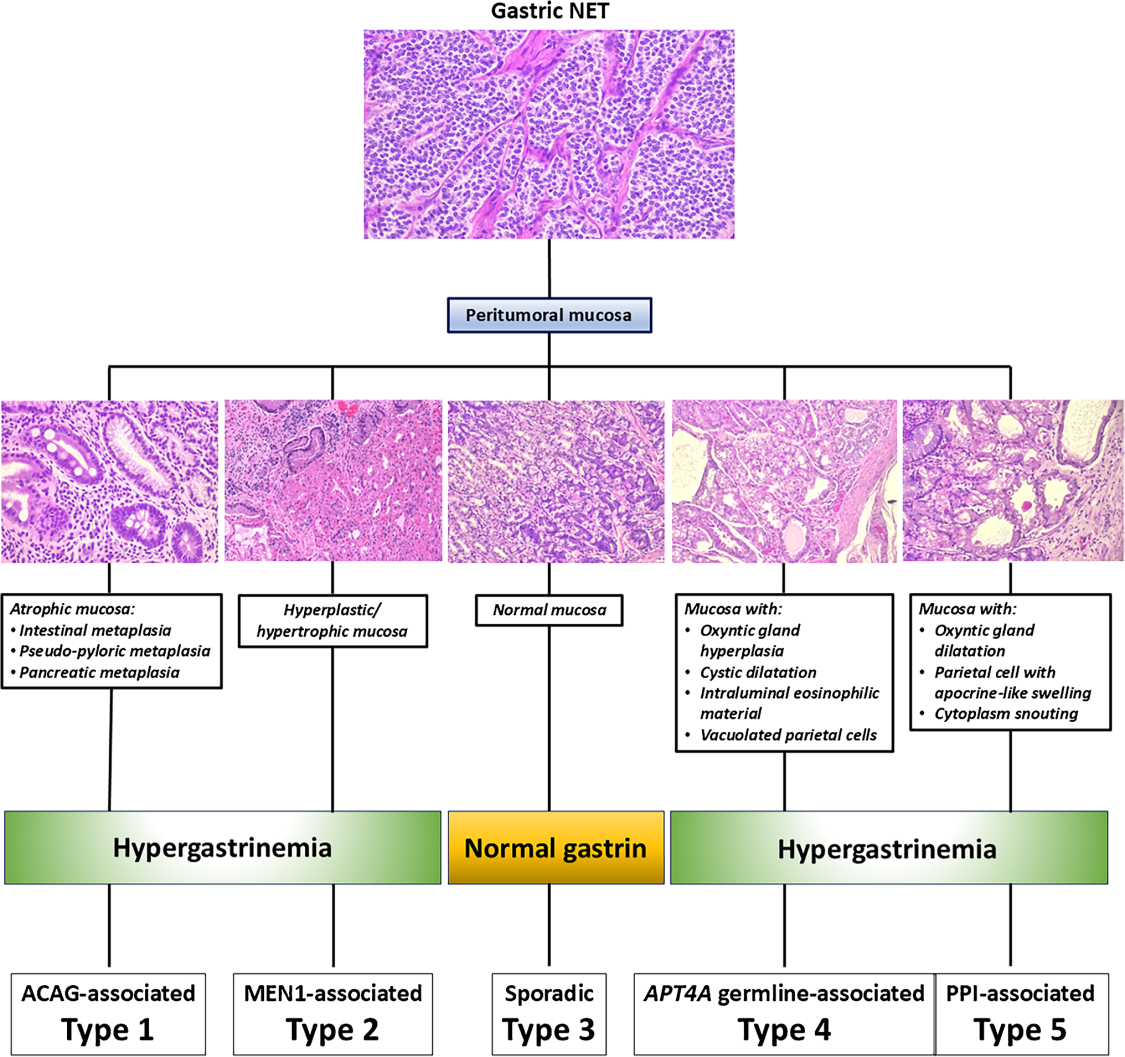
Proposed algorithm for the diagnosis of ECL-cell tumor types combining the morphology of peritumoral mucosa and gastrinemia. ACAG: autoimmune chronic atrophic gastritis. PPI: proton pomp inhibitors

**Fig. 3:**
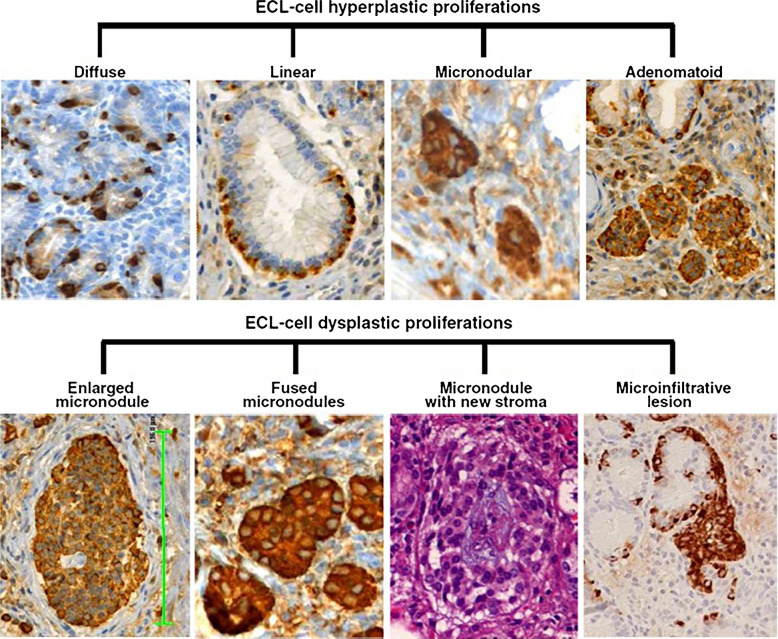
Different types of hyperplastic and dysplastic ECL-cell proliferations. (Published with permission from reference 1)

The key pathologic feature of type 1 ECL-cell NETs is the recognition of atrophic gastritis in the peritumoral mucosa. In full blown cases, oxyntic mucosa is atrophic with intestinal, pseudo-pyloric, and pancreatic metaplasia (Fig. 2). Additional features include muscularis mucosae hyperplasia and a wide spectrum of hyperplastic and dysplastic ECL-cell proliferations due to gastrin stimulation [17]. *ECL-cell hyperplasia* includes four different types of lesions, termed *simple*, *linear*, *micronodular*, and *adenomatoid*, while *ECL-cell dysplasia* includes the following entities: *enlarged (150–500 μm) or fused micronodules*, *micronodule with newly formed stroma* or *micronodules with microinvasion* (Fig. 3) [1]. Simple ECL-cell hyperplasia is characterized by an increase of the number of ECL-cells as scattered elements or collections of less than 5 cells; linear hyperplasia is defined by the presence of sequences of ≥ 5 ECL-cells lying inside the basement membrane of glands; micronodular hyperplasia presents clusters of five or more ECL-cells not exceeding 150 μm in size; adenomatoid ECL-cell hyperplasia is characterized by the collection of five or more micronodules, which are closely adherent to each other but not fused. Dysplastic ECL-cell proliferation defined enlarged micronodule is characterized by the presence of one or more micronodular aggregates with a diameter ranging between 150 and 500 μm; fused micronodule or micronodules in which new stroma formation is observed are considered in the spectrum of dysplastic lesions. The last but not least ECL-cell dysplasia is represented by microinfiltrative lesion defined by the presence of microinfiltration of the lamina propria between glands by neuroendocrine cells (Fig. 3). It is worth noting that, in contrast to micronodular hyperplasia and dysplastic lesions, simple and linear hyperplasia are hardly identifiable in hematoxylin–eosin-stained slides and chromogranin A or VMAT2 immunohistochemistry are useful to detect them. In the antral mucosa gastrin-producing G-cell hyperplasia, which represents the morphological basis of hypergastrinemia, is usually observed [17].

The presence of different ECL-cell proliferations in peritumoral mucosa supports the hypothesis that type 1 ECL-cell tumors develop through the hyperplasia-dysplasia-neoplasia sequence. However, the annual incidence of type 1 ECL-cell NETs in patients with ACAG is low, ranging from 0.1 to 4 per 100 persons [1, 18] suggesting that simple hypergastrinemia is not sufficient for tumor development. The oncogenic role of some growth factors (i.e., TGF-α and bFGF) and mutations of RegIa cooperating with gastrin has been proposed, but needs to be better clarified [19, 20]. The epidemiological information showing the low incidence of ECL-cell NETs in ACAG patients opens an issue about the real oncogenic potential of ECL-cell proliferations when identified in atrophic oxyntic mucosa of ACAG patients without tumor and their need for clinical management. In fact, it has been demonstrated that, among different ECL-cell lesions, only severe ECL-cell hyperplasia, characterized by more than 6 chains of linear hyperplasia per mm, and ECL-cell dysplasia, mainly represented by microinfiltrative lesions, show a significant increased risk for type 1 ECL-cell tumor development [17]. This finding has a practical clinical implication; indeed, the detection of linear or micronodular ECL-cell hyperplasia, almost always observed in biopsies of oxyntic mucosa with ACAG, should not alarm patients and clinicians because the risk of NET development is negligible. For this reason, specific strict endoscopic and histologic surveillance programs are not indicated. In contrast, the observation of severe ECL-cell hyperplasia or dysplastic changes should be considered as potential oncologic risk factors, although only a minority of patients will develop a type 1 ECL-cell tumor during follow-up.

It is of note that patients with chronic atrophic gastritis, including the autoimmune type, show an estimated risk of gastric adenocarcinoma development ranging between 0.53 and 15.24 per 1000 person-year. In addition, gastric adenocarcinoma has been diagnosed in about 23% of patients with type 1 ECL-cell NETs suggesting that patients with ACAG or type 1 ECL-cell NETs should be monitored mainly for the risk of adenocarcinoma development [21, 22]. Interestingly, ACAG-associated adenocarcinomas are often diagnosed at low stage, often display a neuroendocrine component or differentiation, have relatively higher rates of mismatch repair protein (MMR) deficiency, and a high tumor mutational burden (TMB) [23].

Type 1 NETs are relatively indolent with a reported tumor-associated death of 1.07% and a 5-year overall survival reaching 100% [24–26]. Only about 5% of cases metastasize to lymph nodes and these more aggressive tumors generally infiltrate deeply into the wall, confirming that deep invasion is the most important prognostic factor in this ECL-cell tumor subtype [27, 28]. For this reason, an accurate echo-endoscopy is strongly recommended in the diagnostic work-up of type 1 NETs. In cases of early-stage superficial tumors, the “watch and wait” strategy or endoscopic resection are recommended, while more aggressive surgery is discouraged [25]. Indeed, disease-specific survival observed between follow-up endoscopic examinations, with or without endoscopic resection, versus surgical resection is comparable [29]

It is known that type 1 ECL-cell tumors arise as sporadic tumors in the context of ACAG, however, a case associated with pathogenic germline mutation of *BRCA2* has recently.

been described by Zhang et al. who claimed a pathogenic role for the germline *BRCA2* mutation [30].

#### Type 2 ECL-cell NET

Type 2 ECL-cell NETs represented about 8% of ECL-cell NETs in older series [24, 31], but in the more recent ones they seem to be rarer accounting for about 1% of all ECL-cell NETs, due to the increased diagnostic sensitivity for type 1 tumors [4]. There is no gender preference, and the mean age of patients at the time of diagnosis is around 50 years [4, 24, 31]. Type 2 ECL-cell NETs occur in MEN1 patients with hypergastrinemia due to the presence of duodenal or, less commonly, pancreatic gastrinoma. Interestingly, patients with gastrinoma but without MEN1 syndrome do not develop ECL-cell NETs suggesting that the genetic changes present in MEN1 patients are essential to render ECL-cells more susceptible to the proliferative effect of gastrin [32]. Tumors are usually multiple and less than 2 cm in size, mainly confined to the mucosa and submucosa [1, 25] of grade 1 (G1) or grade 2 (G2) [1, 15].

Peritumoral oxyntic mucosa is thickened because of severe hypertrophic hypersecretory gastropathy (Fig. 2) due to gastrinoma-secreted gastrin stimulation. Different types of hyperplastic and dysplastic ECL-cell proliferations are observed in about 85% and 20% of cases, respectively [1, 33]. Hyperplastic ECL-cell proliferations are also observed in about 85% of patients with sporadic gastrinomas but dysplastic lesions are found in only 2% of cases [32].

Lymph node and even distant metastases have been described in about 20% and 10% of patients, respectively [1, 6, 15, 34, 35]. Prognosis is worse than that of patients with type 1 ECL-cell NETs, but better than that of patients with type 3 NETs and largely depends on the management of the concomitant MEN1 syndrome. The surgical resection of the primary gastrinoma may even induce regression of the tumor in some cases [36] The reported 5-year overall survival ranges from 60 to 90% [37].

#### Type 3 NET

Type 3 NETs accounts for about 10% of gNETs and are more frequent in males (M:F ratio: 2.8:1) at mean age of 58 years [1, 4, 24, 37, 38]. They arise in normogastrinemic patients and present as single and large (size around 2 cm) tumors infiltrating the muscular layer in most cases.

Peritumoral oxyntic mucosa is normal (Fig. 2) or shows mild chronic gastritis without hyperplastic or dysplastic ECL-cell proliferations [1, 12]. Since not all tumors with these features show an ultrastructural or immunophenotypical demonstration of ECL-cell differentiation, in the 2019 WHO classification of gastric NENs, it has been suggested to simply call them NETs avoiding the specific term “ECL-cell” [39]. Type 3 NETs can show different proliferative rates, ranging from cases classified as grade 1 (46% of cases) or grade 2/3 (54% of cases) [38]. Lymph node and distant metastases have been reported in about 28% and 25% of patients, respectively [38].

The global 5-year overall survival is < 50%, and different factors including tumor size > 2 cm, vascular and lymphatic invasion, deep invasion of the gastric wall, and the presence of distant metastases strongly influence patient’s outcome, being associated with worse overall survival [26]. Exceptionally long survival was observed in patients with superficial, small, grade 1 tumors [40, 41], while with the increase of grade, tumor size, and depth of infiltration the 5-year survival dramatically decreases under 50% [42, 43]. For these reasons, the European Neuroendocrine Tumor Society (ENETS) and the North American Neuroendocrine Tumor Society (NANETS) have recommended radical resection, with partial or total gastrectomy and regional lymphadenectomy for apparently nonmetastatic lesions > 2 cm [44, 45]. The role of endoscopic resection should be restricted to small (< 1.7 cm) mucosa-confined and low-grade tumors [38].

#### Type 4 ECL-cell NET

Type 4 ECL-cell NETs are very rare and more common in females [46–49]. They are characterized by inactivating mutations of *ATP4A* gene, which encodes the α-subunit of the gastric proton pump leading to achlorhydria, hypergastrinemia, and parietal cell hyperplasia/hypertrophy. In analogy with type 1 NETs, compensatory gastrin hypersecretion represents a strong stimulus for ECL-cell proliferation. Type 4 ECL-cell NETs have also been reported as hereditary disease since germline mutation of the *ATP4A* was detected in consanguineous members of a family where five subjects presented GNETs [47].

Tumors are small and multiple and peritumoral oxyntic mucosa shows ECL-cell proliferations and distended fundic glands containing inspissated proteinaceous eosinophilic material and lined by large hypertrophic parietal cells, some of them with vacuolated cytoplasm showing protrusions of luminal surface (Fig. 2) [46, 49].

Before its definitive identification on the basis of the underlying genetic defect [47], this ECL-cell NET subtype was probably already reported in two hypergastrinemic patients with multiple gastric NETs arising in oxyntic hyperplastic mucosa, without MEN1 syndrome or gastrinoma, in 1995 and 2005 by Ooi et al. and Abraham et al. Interestingly, in one of these patients, peritumoral oxyntic mucosa showed parietal cells with large cytoplasm containing poorly developed intracytoplasmic canaliculi and vesico-tubular profiles and numerous mitochondria. These ultrastructural findings suggested an intrinsic HCl secretion abnormality, likely responsible for the patient's hypergastrinemia [50, 51]. Although molecular analysis was not performed in these two cases, they seem to represent the first description of type 4 ECL-cell NET.

Type 4 ECL-cell NETs are mostly stage I or II and regional lymph node metastases have been reported. Due to their rarity and recent recognition, 5-year survival rates are unknown.

#### Type 5 ECL-cell NET

Since the first description in 1998 of a gastric NET detected during long-term anti-ulcer therapy with H2 receptor antagonist and proton pump inhibitor (PPI) [52], the potential role of long-term PPI therapy as risk factor for developing gastric NETs has largely been discussed [53]. Recently published data demonstrated that a minority of patients treated with high doses of PPI for more than 10 years show a low risk to develop indolent gastric NETs [53–56], suggesting caution in using PPI therapy for long periods of time, especially when starting in young subjects [53]. Hypergastrinemia consequent to the inhibitory effects of therapy on acid secretion certainly plays a pathogenetic role. However, underlying conditions or genetic factors such as a PPI-metabolizer phenotype with slow metabolizers or chronic *Helicobacter pylori* gastritis with or without mucosa atrophy seem to be required for NET development [53].

ECL-cell NETs arising in patients treated long-term with PPIs have recently been defined as type 5 ECL-cell NETs [13]. They are more frequent in males, arise in the oxyntic mucosa and present as small polyps or nodules; 30% of cases are multifocal [54–56]. In most instances, they are G1 or G2 tumors and are confined to mucosa/submucosa.

Peritumoral oxyntic mucosa shows features generally observed in PPI-treated patients including oxyntic gland dilatation, parietal cell with apocrine-like swelling and cytoplasmic snouting (Fig. 2). ECL-cell hyperplasia is the rule.

Although lymph node and distant metastases have rarely been described, type 5 ECL-cell NETs are indolent with an excellent prognosis [54–56]. For this reason, they need to be separated from type 3 NETs, which are more aggressive. This differential diagnosis may not be easy because peritumoral oxyntic mucosa may appear quite normal, although PPI-related alterations can be found if accurately searched for. Dosing gastrin serum levels, which are high in these patients, may support the correct diagnosis.

#### Proposed simplified classification of ECL-cell NETs

The clinico-pathological classification of ECL-cell NETs into five subtypes, as described here, provides a comprehensive and meaningful framework for understanding their pathogenesis and predicting prognosis. Nevertheless, in daily clinical practice, a simplified approach—based on gastrin serum levels and the status of gastric acid secretion—may be preferable to expedite patient management.

**Fig. 4:**
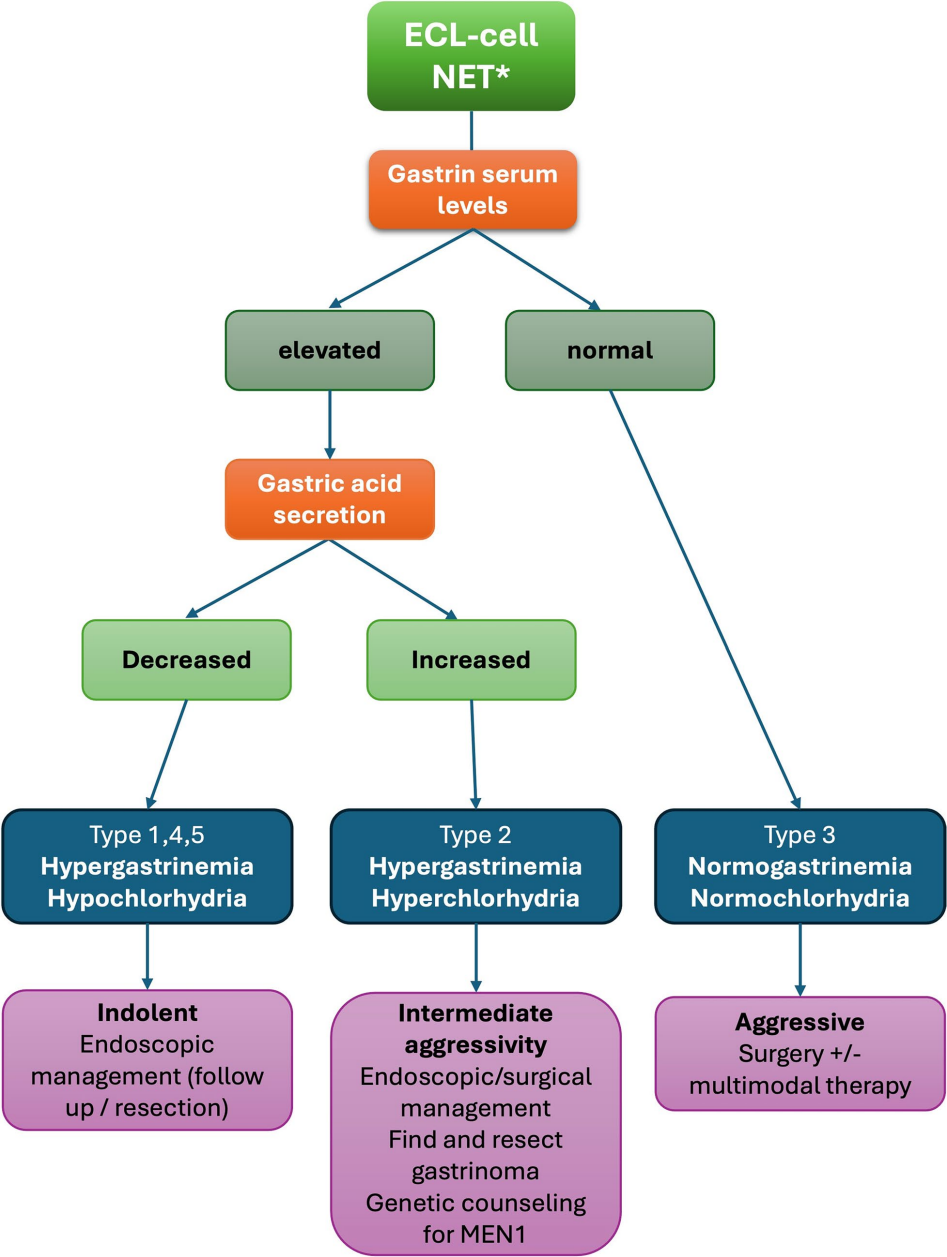
Diagnostic algorithm for a simplified classification of ECL-cell NETs. *: G-cell, D-cell, and EC-cell NETs are excluded

Indeed, types 1, 4, and 5, all associated with *hypergastrinemia and achlorhydria* (albeit for different reasons), can generally be managed according to the depth of wall invasion; when superficial, they may be treated endoscopically [25, 29]. By contrast, type 2 ECL-cell NETs, which are associated with *hypergastrinemia and hyperchlorhydria*, are intrinsically more aggressive than their hypergastrinemic hypo/achlorhydric counterparts and require a careful search for underlying gastrinoma, which should be surgically removed. When appropriate, genetic counseling for MEN1 should also be offered. Finally, type 3 NETs, characterized by *normogastrinemia and normochlorhydria*, are aggressive lesions best managed surgically, with multimodal therapy considered in advanced disease [44, 45]. Figure 4 illustrates this simplified classification algorithm.

In summary, in a diagnostic setting, when the pathologist encounters a gNET on a small biopsy without relevant clinical information, adding a comment to the report recommending assessment of gastrin serum levels and gastric secretion status may provide the clinician with the tool to quickly obtain the key information needed for an initial management decision. In addition, the suggestion to repeat endoscopy to provide peritumoral mucosa may be recommended.

#### Serotonin-producing EC-cell tumors

Serotonin-producing EC-cell gNETs are very rare if compared with the distal intestinal counterparts, with only a few cases reported in the literature [57, 58]. They are usually non-functioning but can be associated with the classic carcinoid syndrome [58]. Due to their rarity, no reliable data about epidemiology and patients’ outcome is available.

**Fig. 5:**
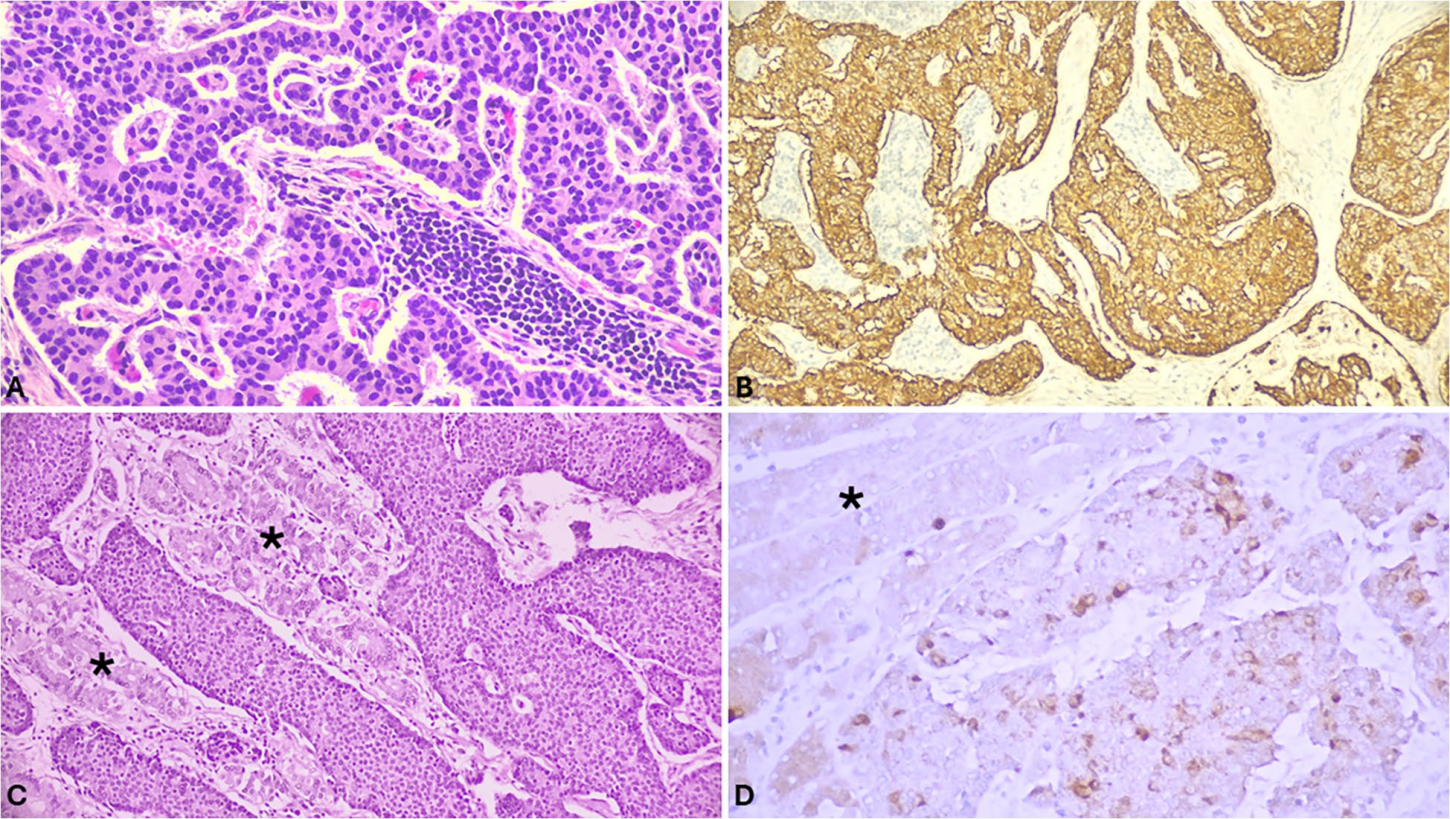
**A** Antral gastrin-producing G-cell NET showing a typical trabecular-gyriform architecture. **B** Tumor cells are diffusely and strongly positive for gastrin. **C** Sertonin-producing EC-cell NET of the stomach composed of cells forming nests with peripheral palisading. Residual gastric glands are identified by the *. **D** Tumor cells are positive for serotonin

Serotonin-producing cell gNET morphology overlaps that of ileal EC-cell NETs, featuring rounded nests with peripheral palisading of uniform cells with intense eosinophilic granular cytoplasm (Fig. 5). By immunohistochemistry, they are positive for serotonin and show an intense nuclear staining for CDX2.

#### Gastrin-producing G-cell tumors

G-cell NETs are usually located in the antral mucosa, most frequently in proximity to the pylorus, and are extremely rare. They are almost always non-functioning, but in a minority of cases cause the Zollinger–Ellison syndrome; for these functioning cases the term “gastrinoma” is used.

These tumors appear as small mucosal/submucosal nodules, composed of uniform polygonal cells with scant cytoplasm arranged in thin trabecular and gyriform structures (Fig. 5), although some cases grow in solid nests [1]. Tumor cells are intensely positive for gastrin and CDX2 nuclear stain is weaker than in serotonin-producing EC-cell NETs.

Most cases are limited to antral wall, although nodal metastases have been described [59]. The prognosis is generally excellent, despite the presence of histological signs of aggressiveness including muscular infiltration or lymph node metastasis in about 18% of cases [24].

#### Somatostatin-producing D-cell tumors

D-cell NETs of the stomach are exceptionally rare and mostly are incidental findings during gastric endoscopy, appearing as antral nodules, although they can also be found in the corpus mucosa. These are usually nonfunctioning tumors, not associated with symptoms of the full blown somatostatinoma syndrome; however, they can present with mass-related nonspecific symptoms such as gastric bleeding [1, 24, 60]. Tumors are composed of well-differentiated monomorphic neuroendocrine cells that are positive for general neuroendocrine markers and somatostatin.

Due to their rarity the prognosis of these NETs is unknown, but the fact that in most cases they are small and superficial suggests that their prognosis is excellent.

### Gastric NECs (gNEC)

gNECs are poorly differentiated carcinomas showing neuroendocrine morphology and expressing general neuroendocrine markers. They represent about 15–20% of all gNENs and account for about 20% of all digestive NECs. They are more frequently diagnosed in males (male/female ratio of 2:1) at an average age of 65 years (range 41–76 years) [4, 24, 61]. Similarly to non-neuroendocrine gastric cancer, patients present with nonspecific symptoms including dyspepsia, weight loss, gastric bleeding, anemia, and abdominal pain, especially when the tumor is ulcerated.

**Fig. 6:**
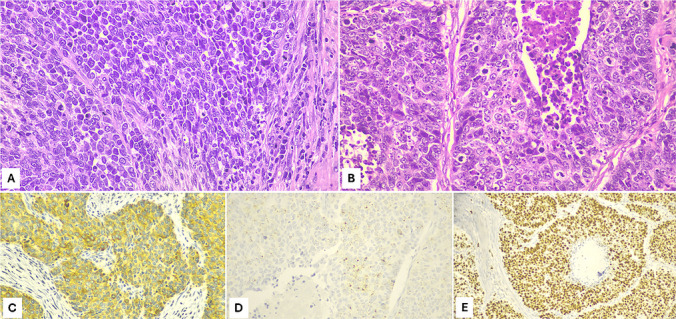
Gastric NECs can be separated into small cell or large cell subtypes depending on the morphology of tumor cells. Small cell NECs are composed of small to medium-sized (2–4 times of lymphocyte size), round to oval cells with scant cytoplasm and hyperchromatic nuclei without nucleoli (**A**), while large cell NECs are composed of large cells with vesicular nuclei showing prominent nucleoli and abundant eosinophilic cytoplasm. In this field, tumor necrosis is well evident (**B**). NECs are diffusely immunoreactive for synaptophysin (**C**), while chromogranin A can be focally positive with a paranuclear dot-like pattern (**D**). The Ki67 proliferation index is high, and frequently higher than 70% (**E**).

gNEC more frequently arises in the cardial or antral region and presents as a single large and ulcerated mass [7, 24, 34]. Histologically, these neoplasms are composed of poorly differentiated cells, growing in sheets, nests or organoid structures, with extensive necrosis, high mitotic count (> 20 mitoses/mm^2^), high Ki67-proliferation index (always > 20%, although frequently > 70%), as well as perineural and vascular invasion. Based on cell morphology, NECs are separated into small cell and large cell subtypes: small cell NECs are composed of small to medium-sized (< 3 resting lymphocyte size), round to oval cells with a barely visible rim of cytoplasm and hyperchromatic nuclei without nucleoli. Large cell NECs are composed of large cells with vesicular nuclei, prominent nucleoli and abundant eosinophilic cytoplasm (Fig. 6).

Although morphology is quite characteristic, the diagnosis of gastric NEC needs to be supported by immunohistochemistry because there are some non-neuroendocrine neoplasms such as poorly differentiated adenocarcinoma, lymphoma, or mesenchymal proliferations that show similar morphological features. In this context, the demonstration of neuroendocrine phenotype is mandatory. It is important to underline that CD56, PGP9.5, and neuron specific enolase (NSE) immunohistochemistry should not be used since these markers are highly nonspecific [62, 63]. Conversely, the use of at least two antibodies among synaptophysin, chromogranin A and INSM1 is the best approach (Fig. 6). The use of only one marker is discouraged because their sensitivity and specificity are not 100%. Synaptophysin shows high sensitivity but lower specificity and may also be expressed in some non-neuroendocrine neoplasms including sarcomas. Conversely, chromogranin A is highly specific but shows lower sensitivity. This is explained by the fact that NECs produce a low number of secretory granules where chromogranin is stored together with the hormone. For this reason, chromogranin A can be only focally positive or even negative [62, 63]. INSM1 is a recently introduced marker in the diagnostic work-up that, while highly sensitive and specific, is positive in about 92% of gastric NECs and therefore does not identify all cases [64]. It is very important to recall that gastric NECs can be aberrantly positive for CDX2 and TTF1 due to a phenomenon designated “transcription factor lineage infidelity”, and thus, expression of these markers in NECs should not be misinterpreted as metastases from intestinal or lung primary NECs [63].

Immunohistochemistry also plays a role for the differential diagnosis between NET G3 and large cell NEC, both showing high Ki67 proliferative index and sometimes similar morphological features. In this context, p53 and, especially, Rb immunohistochemistry is particularly useful since their expression reflects the molecular background. Indeed, NECs are characterized by *TP53* and *RB1* mutations which are reflected in the strong and diffuse (“mutated pattern”) or totally absent (“null pattern”) expression of p53 and global loss of Rb immunoreactivity, respectively [63, 65]. However, it is worth noting that a small percentage of NET G3 can show altered expression of both proteins [66], so it is prudent to evaluate their expression in the context of morphology to reduce the risk of misinterpretation. Immunohistochemistry can also play a prognostic role since the identification of mismatch repair (MMR) proteins deficiency allows to identify patients with better prognosis [67, 68]. In addition, the combined evaluation of tumor-infiltrating lymphocytes (using CD3 antibodies), MMR proteins (using hMLH1, hPMS2, hMSH2, and hMSH6 antibodies) and PD-L1 status may be useful to select patients who can benefit from immunotherapy [69].

gNECs are aggressive cancers with poor prognosis. Survival time is usually measured in months; no statistically significant survival difference between small cell and large cell subtypes has been proven. The prognosis is influenced by several factors including tumor of location, pathological T stage, presence of lymph node and/or distant metastases and MMR protein status [67, 70].

Extremely rare cases of gastric Merkel cell carcinomas have been reported and at least one of them was demonstrated to be primary of the stomach and not a gastric metastasis from a cutaneous cancer [71]. Primary gastric Merkel cell carcinoma showed the same morphological and immunohistochemical features including CK20 and Merkel cell polyomavirus (MCPyV) expression of skin counterpart. Consideration of this rare entity by immunohistochemistry is recommended when morphology is fitting.

### Gastric MiNENs (gMiNENs)

gMiNENs are mixed epithelial neoplasms in which a neuroendocrine component is combined with a non-neuroendocrine carcinoma, each of which is morphologically and immunohistochemically recognizable and represents ≥ 30% of the tumor mass, although this cut-off has been arbitrarily chosen and does not have a biological and clinical significance [5]. The unproven clinical role of this cut-off has also been recently suggested by the fact that even minimal NEC component in an adenocarcinoma may exert a significantly adverse biological influence [70].

gMiNENs account for about 6–20% of all digestive MiNENs, are more common in males, and the mean age at diagnosis is in the fifth and sixth decades [72]. As gastric NECs, patients present with nonspecific symptoms and tumors macroscopically resemble gastric adenocarcinoma presenting as ulcerated or polypoid large lesions [72].

**Fig. 7:**
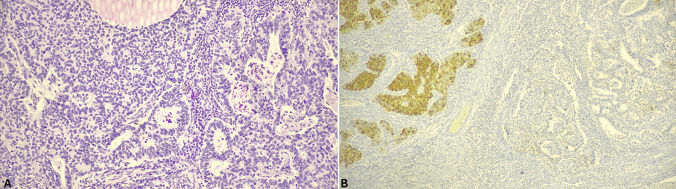
MiNENs are characterized by the presence of two recognizable components (**A**). In most cases the non-neuroendocrine component is represented by adenocarcinoma (right part of A), while the neuroendocrine component by NEC shows solid architecture (upper left part of B). Neuroendocrine markers such as synaptophysin are useful to confirm the diagnosis being positive in the neuroendocrine component and negative in the adenocarcinoma one (**B**)

gMiNENs are usually composed of adenocarcinoma associated with NEC (Fig. 7), which have been previously termed mixed adenocarcinoma-neuroendocrine carcinoma (MANEC). From a practical point of view, when a gastric neoplasm presents two different components of which one with the typical morphology of gastric adenocarcinoma or related subtypes (signet ring cell, papillary, mucinous, etc.…) and the other showing solid or organoid structure with extensive necrosis and high mitotic count, a possible MiNEN should be considered. Immunohistochemistry plays a crucial role demonstrating the neuroendocrine differentiation of this second component [72, 73]. It is worth noting that the Ki67-proliferative index of the NEC component has a prognostic meaning since Ki67 < 55% seems to be associated with better survival [68]. As with NECs, MMR-deficient MiNENs are associated with better survival than MMR-proficient MiNENs [67]. gMiNENs composed of adenocarcinoma and NET have been described as well, although rarer than MiNENs in which the neuroendocrine component is represented by NEC [74]. Interestingly, some cases were observed in patients with ACAG [73]. Mixed adenocarcinoma-NETs are composed of areas of tubular, papillary, or mucinous adenocarcinoma and areas of NET that can be of G1, G2 or G3 grade.

The prognosis of adenocarcinoma-NEC MiNENs overlaps that of pure NEC [68], while adenocarcinoma-NET cases seem to have a better survival.

Related to gMiNENs, rare gastric mixed neoplasms composed of adenoma and NET have been reported and defined Mixed Adenoma-NET (MANET) [75, 76]. However, this category is not formally considered a MiNEN subtype since the non-neuroendocrine component is not malignant. Independently of nosological and terminological definitions, this entity can be encountered in routine practice and needs to be identified and distinct from conventional MiNENs because it shows an excellent behavior.

These tumors appear as flat or elevated lesions with sizes ranging from 0.8 to 4.4 cm, located in the body mucosa, which generally exhibits chronic metaplastic atrophic gastritis [76]. The glandular component is represented by intestinal-type adenoma, while the NET component, generally located in the middle and deep part of the polyp, shows the typical morphologic features of NET and can show different grades based on both mitotic index and Ki67-proliferation index.

The prognosis of MANETs is excellent with no reported tumor-related death [75].

## Concluding remarks

Using the WHO classification scheme, gNENs are separated into three different entities (NET, NEC, and MiNEN), which show specific morphologic, immunohistochemical, clinical, and prognostic features. Furthermore, NETs are separated into three proliferative grades (G1, G2, and G3).

In ECL-cell NETs, the clinical and prognostic power of proliferative grade is less relevant than in other sites such as the pancreas. Indeed, for a clinically relevant classification of ECL-cell NETs, there needs to integrate proliferative grade with the clinicopathologic background. A complete assessment, that also takes into account pathogenetic mechanisms, includes the morphology of peritumoral oxyntic mucosa, the presence of antral G-cells hyperplasia, the presence of hypergastrinemia, the setting of MEN1 syndrome, the presence of hypo/achlorhydria, the presence of anti-parietal cells and/or intrinsic factor autoantibodies, the presence of megaloblastic anemia, mutational status of *ATP4A* gene and long-standing treatment with PPI. This integrated approach separates ECL-cell NETs into five subtypes with different clinical presentation and behavior. However, in daily practice, where small biopsy specimens and limited clinical information are commonplace, information about gastrin serum levels and gastric secretion status may be sufficient for the initial management of the patient. On this basis, we hereby propose a simplified classification of ECL-cell NETs that distinguishes NETs related to *hypergastrinemia and achlorhydria* (that bear very good prognosis) from those arising in the context of *hypergastrinemia and hyperchlorhydria* (that have an intermediate prognosis) and those with *normogastrinemia and normochlorihydria* (that are the most aggressive ones).

Antral NETs including gastrin-, somatostatin-, and serotonin-producing tumors are very rare and seem to show indolent behavior.

NECs and MiNENs (especially mixed adenocarinoma-neuroendocrine carcinomas) are aggressive cancers that need to be separated from NETs due to their different therapeutic approach.

## Data Availability

Not applicable. This is a review article.
